# Endoscopic resections for superficial esophageal squamous cell epithelial neoplasia: focus on histological discrepancies between biopsy and resected specimens

**DOI:** 10.1186/s12876-021-01694-9

**Published:** 2021-03-09

**Authors:** Lang Yang, Hua Jin, Xiao-li Xie, Yang-tian Cao, Zhen-hua Liu, Na Li, Peng Jin, Yu-qi He, Jian-qiu Sheng

**Affiliations:** 1 Department of Gastroenterology, The Seventh Medical Center of Chinese PLA General Hospital, No. 5 Nanmencang, Dongcheng District, Beijing, 100700 China; 2 Department of Pathology, The Seventh Medical Center of Chinese PLA General Hospital, Beijing, China

**Keywords:** Esophageal neoplasia, Histology, Biopsy, Endoscopy

## Abstract

**Background:**

Endoscopic resection has been used for high-grade intraepithelial neoplasia (HGIN) and superficial esophageal squamous cell carcinoma (ESCC) with limited risk of lymph node metastasis. However, some of these lesions cannot be accurately diagnosed based on forceps biopsy prior to treatment. In this study we aimed to investigate how to solve this histological discrepancy and avoid over- and under-treatment.

**Methods:**

The medical records of patients with superficial esophageal squamous cell neoplasia who underwent endoscopic resection at our hospital from January 2012 to December 2019 were reviewed retrospectively. The histological discrepancy between the biopsy and resected specimens was calculated and its association with clinicopathological parameters was analyzed.

**Results:**

A total of 137 lesions from 129 patients were included. The discrepancy rate between forceps biopsy and resected specimens was 45.3% (62/137). Histological discrepancy was associated with the histological category of the biopsy (*p* < 0.001). In addition, 17 of the 30 (56.7%) biopsies that was diagnosed as indefinite/negative for neoplasia or low-grade intraepithelial neoplasia were upgraded to HGIN or ESCC after resection. The upgrade was due to lesion size ≥ 10 mm (*p* = 0.002) and type B intrapapillary capillary loops (*p* < 0.001). Moreover, 34 of the 83 biopsies that were diagnosed with HGIN were upgraded to ESCC after resection, which was related to lesion size (*p* = 0.001), location (*p* = 0.018), and pink color sign (*p* = 0.002).

**Conclusions:**

Histological discrepancy between forceps biopsy and resected specimens is common in clinical practice. Recognizing the risk factors for each histological category of biopsy may reduce these discrepancies and improve clinical management.

**Supplementary Information:**

The online version contains supplementary material available at 10.1186/s12876-021-01694-9.

## Background

Esophageal cancer is the seventh most common cancer worldwide [1]. In China, esophageal squamous cell carcinoma (ESCC) is the dominant histological type (approximately 90%), and accounts for more than half of new global cases [2, 3]. The prognosis of ESCC depends on the tumor stage as the five-year-survival rate can exceed 90% for early-stage cancer, which is only 10–20% for advanced cancer [4]. Endoscopy with forceps biopsy is the standard procedure for early diagnosis, and it has been shown to decrease incidence and mortality of ESCC [5]. Detected lesions are managed based on histological evaluation of biopsy fragments according to the Vienna classification [6]. Superficial ESCC with low risk of lymph node metastasis and high-grade intraepithelial neoplasia (HGIN) are indications for endoscopic resection [7–10]. Thus, careful selection of patients prior to treatment is essential for avoiding under- or over-treatment.

However, histological discrepancies between forceps biopsy and resected specimens in superficial esophageal squamous cell neoplasia (SESCN) is not uncommon [11, 12]. Jin et al. [12] reported that the diagnostic accuracy rate was 51% for pretreatment biopsy. A study in the United Kingdom(UK) showed that after endoscopic resection, the histological grade was changed in 79.2% of SESCNs [13]. Korean scholars have reported the histological discrepancy rate to be 34.5%, related to upper tumor location and tumor area/biopsy [14]. However, these studies used different standards to categorize the histology and no consensual conclusions were drawn regarding management of histological discrepancy.

In this study, we retrospectively analyzed the clinicopathological features of endoscopically resected SESCN in our center and investigated the risk factors of histological discrepancies between forceps biopsy and resected specimens.

## Methods and patients

### Patients

The medical records of consecutive patients with SESCN who underwent endoscopic resection at our center from January 2012 to December 2019 were reviewed retrospectively. SESCN consists of superficial ESCC and precancerous lesions. Precancerous lesions are classified as either low-grade intraepithelial neoplasia (LGIN) or HGIN. Superficial ESCC is defined as tumor cells limited to the mucosal and submucosal layers. The exclusion criteria for this study were as follows: (1) patients who received radiotherapy and photodynamic therapy prior to endoscopic resection, (2) patients transferred from a local hospital who did not have a biopsy performed at our center, and (3) patients without magnifying endoscopy or chromoendoscopy. This study was designed in accordance with the ethical standards of the institutional research committee and with the 1964 Helsinki Declaration and its later amendments or comparable ethical standards.

### Histological evaluation

For forceps biopsy and endoscopic resection, the specimen was fixed, embedded, and cut. The hematoxylin–eosin-stained slides were reviewed by two pathologists blindly to clinical information (H. J. and XL. X.). When the pathological diagnosis was not consistent between the two reviewers, an agreement was reached through discussion. The pathological diagnosis of ESCC and squamous cell intraepithelial neoplasia was based on the fifth edition of World Health Organization (WHO) classification of digestive tumors [15]. Briefly, LGIN involves neoplastic cells in only the lower half of the epithelium with only mild cytological atypia and HGIN involves neoplastic cells in more than half of the epithelium or severe cytological atypia regardless of the extent of epithelial involvement. When LGIN, HGIN and/or ESCC coexisted, the diagnosis was made according to the highest grade involved. En bloc resection was defined as an intact lesion that was removed under endoscopy. R_0_ resection was defined a lesion that was resected en bloc and negative for vertical and lateral margins.

### Endoscopic characteristics

Endoscopy examinations were carried out using GIF-H260, GIF-Q260J, GIF-HQ290 and GIF-H260Z (Olympus Corporation, Tokyo, Japan). The gross type of lesion was classified according to the Paris classification and categorized as elevated (type 0-I and 0-IIa), flat (type 0-IIb), and depressed (type 0-IIc and 0-III) [16]. The location of the lesion was divided into the upper, middle, and lower esophagus according to a previous paper [17]. The upper esophagus was defined as the 5 cm length of esophagus distal to the upper esophageal sphincter, the lower esophagus was defined as the 5 cm length of esophagus proximal to the lower esophageal sphincter, and the remaining region between the upper and the lower esophagus was defined as the middle-esophagus. The color of the lesion was classified as reddish, whitish, or no obvious change. The pink color sign was determined 2–3 min after spraying 1% percent Lugol’s iodine solution[18]. Intrapapillary capillary loops (IPCLs) was classified as type A or type B according to the Japan Esophageal Society Classification of Magnified Endoscopy, and background color was assessed under narrow banding imaging magnifying endoscopy according to the reference [19]. In addition, the area of tumor or biopsy tissue was calculated using the formula for an ellipse (area = 3.14 × length × width/4). The length and width of the tumor was from the endoscopic record, and the size of biopsy tissue was measured under microscopy based on the tissue in slides.

### Statistical analysis

IBM SPSS Statistics 26 (SPSS, Inc., Chicago, USA) was used for statistical analysis. The categorical data were analyzed using the chi-square or Fisher’s exact test. Normally distributed continuous variables were analyzed using the Student *t* test, while, abnormally distributed continuous variables were analyzed using the Mann–Whitney *U*-test. A *p *value < 0.05 was regarded as statistically significant.

## Results

### Baseline patient characteristics

A total of 137 lesions from 129 patients were included in this study (Table 1). The patients were aged 62.4 ± 7.8 years (range, 43- 82) and were predominantly male (67.1%). The lesions were 21.9 ± 15.1 mm (from 3 to 100 mm) in size, and 122 of the lesions (89.1%) involved < 50% of the circumferential extent of the esophagus. A total of 105 lesions (76.6%) were located in the middle esophagus, 19 (13.9%) in the lower esophagus, and 13 (9.5%) in the upper esophagus. The lesions were mostly in reddish in color (72.3%) and flat in appearance (70.8%). A total of 125 of the lesions (91.2%) were removed by endoscopic submucosal dissection (ESD) and the rest were multiband mucosectomy (MBM). The en bloc resection rate was 97.1% (133/137), and the R_0_ resection rate was 94.9% (130/137). Perforations occurred in four patients (2.9%) during endoscopic resection and were closed using clips. Delayed bleeding during hospitalization occurred in two patients (1.5%) and was treated by endoscopic hemostasis. Based on resected specimens, 2 lesions (1.6%) were histologically diagnosed as negative for neoplasia, 16 (11.6%) as LGIN, 60 (43.8%) as HGIN, and 59 (43%) as ESCC. Twelve of the 59 patients diagnosed with ESCC (20.3%) had submucosal cancer.

**Table 1:** Clinicopathological features of patients with superficial squamous cell neoplasia (N = 137)

Variables	Results
Age, years, mean ± SD	62.4 ± 7.8
Age in subgroups, years, n (%)	
< 50/ ≥ 50, < 60/ ≥ 60, < 70/ ≥ 70	9(6.6)/37(27.0)/68(49.6)/23(16.8)
Sex, n (%)	
Male/female	92(67.1)/45(32.9)
Lesion size, mm, mean ± SD	21.9 ± 15.1
Size in subgroups (mm), n (%)	
< 10/ ≥ 10, < 20/ ≥ 20, < 30/ ≥ 30	27(19.7)/43(31.4)/30(21.9)/37(27.0)
Circumferential extent of lesions, n (%)	
< 50%/50–75%/ > 75%	122(89.1)/12(8.7)/3(2.2)
Location in esophagus, n (%)	
Upper/middle/lower	13(9.5)/105(76.6)/19(13.9)
Color under white light endoscopy, n (%)	
Reddish/whitish/no obvious change	99(72.3)/23(16.8)/15(10.9)
Gross type, n (%)	
Elevated/flat/depressed	11(8.0)/97(70.8)/29(21.2)
Treatment, n (%)	
ESD/MBM	125(91.2)/12(8.8)
En bloc resection, n (%)	133(97.1)
R_0_ resection, n (%)	130(94.9)
Perforation, n (%)	4(2.9)
Stricture, n (%)	10(7.3)
Delayed bleeding, n (%)	2(1.5)
Final histology, n (%)	
Negative for neoplasia/LGIN/HGIN/ESCC	2(1.6)/16(11.6)/60(43.8)/59(43)
Mucosal cancer/Submucosal cancer	47(79.7)/12(20.3)

ESD = endoscopic submucosal dissection, MBM = multiband mucosectomy, LGIN = low-grade intraepithelial neoplasia, HGIN = high-grade intraepithelial neoplasia, ESCC = esophageal squamous cell carcinoma, SD = standard deviation

### Histological discrepancy between forceps biopsy and resected specimens

The final histological categories of the 137 lesions according to biopsy diagnosis are listed in Table 2. The discrepancy rate between forceps biopsy and resected specimens was 45.3% (62/137), and 17 of the 119 (14.3%) ESCC and HGIN diagnoses were under diagnosed as LGIN, or indefinite/negative for neoplasia. For biopsy diagnosed LGIN, the discrepancy rate was 50% (8/16), with 7 of 16 (43.7%) LGIN-diagnosed biopsies upgraded to HGIN or ESCC according to resected specimens. For the HGIN lesions diagnosed on biopsy, the discrepancy rate was 45.8% (38/83), and 34 of the 83 lesions (41.0%) were upgraded to ESCC. The discrepancy rate for biopsy confirmed ESCC is 8.4% (2/24), with one lesion downgraded to HGIN and for another small lesion, neoplastic cells could not be found in the resected specimen. We then investigated the factors associated with histological discrepancy. We found that it was significantly associated with the histological category of the biopsy (*p* < 0.001) (Additional file 1: Table S1), but not associated with age (*p* = 0.128), sex (*p* = 0.550), lesion color (*p* = 0.356), location size (*p* = 0.599) and location (*p* = 0.086), gross type (*p* = 0.353), pink color sign (*p* = 0.791), IPCL (*p* = 0.945), background color (*p* = 0.833), number of biopsies performed (*p* = 0.364), biopsy tissue area (*p* = 0.261), median tumor area(*p* = 0.782) and median tumor area/number of biopsies(*p* = 0.556), or duration of time between biopsy and resection (*p* = 0.654) (Additional file 1: Table S1).

**Table 2:** Histology of the lesions based on forceps biopsy and resected specimens

Pre-treatment biopsy	Histology based on resected specimen n (%)			
	Negative for neoplasia (n = 2)	LGIN (n = 16)	HGIN (n = 60)	ESCC (n = 59)
Negative for neoplasia (n = 3)	0 (0)	0 (0)	2 (66.7)	1 (33.3)
Indefinite for neoplasia (n = 11)	0 (0)	4 (36.4)	7 (63.6)	0 (0)
LGIN (n = 16)	1 (6.3)	8 (50)	5 (31.2)	2 (12.5)
HGIN (n = 83)	0 (0)	4 (4.8)	45 (54.2)	34 (41.0)
ESCC (n = 24)	1 (4.2)	0 (0)	1 (4.2)	22 (91.7)

LGIN = low grade intraepithelial neoplasm, HGIN = high grade intraepithelial neoplasm, ESCC = esophageal squamous cell carcinoma

### Histological upgrade to HGIN and ESCC in patients diagnosed as negative/indefinite for neoplasia, and LGIN on biopsy

**Fig. 1:**
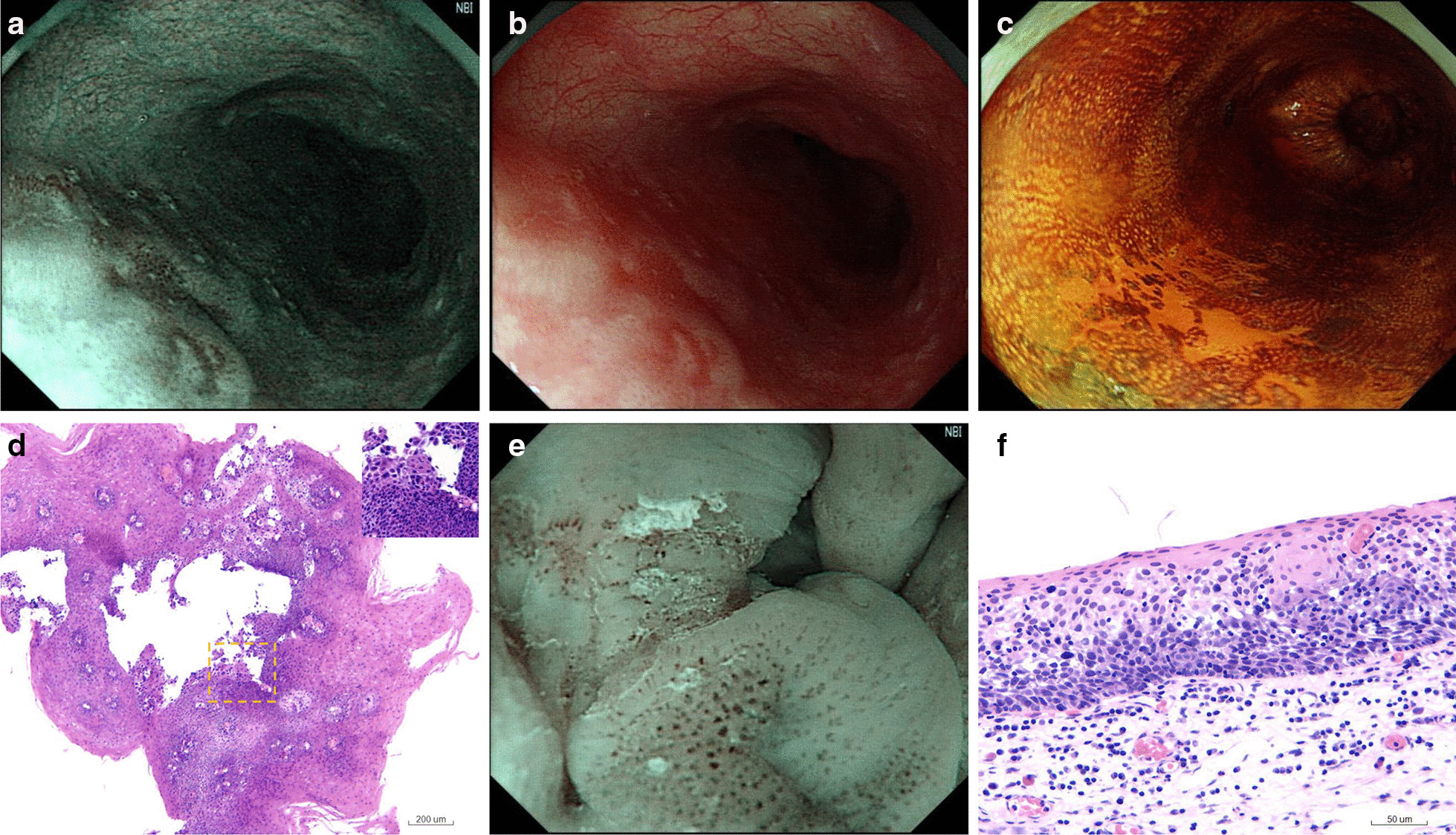
A patient who was diagnosed as indefinite for neoplasia on biopsy and subsequently upgraded to HGIN after resection. **a** A brownish area is observed in the middle esophagus; **b** the lesion is flat and reddish under white light endoscopy; **c** map-like unstained area is observed after 1% iodine spraying but negative for pink color sign. **d** One biopsy is taken from unstained area and is diagnosed as indefinite for neoplasia; **e** magnifying endoscopy shows type B1 IPCL; and **f** the lesion is diagnosed as HGIN after resection

For the 30 lesions that were diagnosed as negative/indefinite for neoplasia or LGIN on biopsy, 17 (56.7%) were upgraded to HGIN or ESCCs after resection. Perforation occurred in one patient during endoscopic procedure and was closed using clips, after which no delayed bleeding occured. One lesion was piecemeal resected by MBM with indefinite lateral margin, and no residual lesion was observed during follow-up. For the remaining 16 lesions, curative resections were achieved. Histological upgrades to HGIN or ESCC were associated with lesion size ≥ 10 mm (*p* = 0.002) and type B IPCL (*p* < 0.001), but were not significantly associated with age (*p* = 0.465), sex (*p* = 0.264), lesion location (*p* = 0.971), color (*p* = 0.167), gross type (*p* = 0.379), pink color sign (*p* = 0.613), or brownish background (*p* = 0.138) (Table 3). The positive and false predictive values of lesion size ≥ 10 mm for diagnosing HGIN and ESCC were 78.9% and 81.8% respectively. Interestingly, none of the lesions with type A IPCL were upgraded to HGIN or ESCC. The positive and false predictive values of type B IPCL for diagnosing HGIN and ESCC were 85.0% and 100.0%, respectively. A representative case of biopsy-diagnosed indefinite neoplasia upgraded to HGIN is shown in Fig. 1.

**Table 3:** Factors associated with HGIN and ESCC in patients diagnosed as negative/indefinite for neoplasia or LGIN on biopsy

Variables	Total (n = 30)	Final histology		*p* value
		No-IN/LGIN (n = 13)	HGIN/ESCC (n = 17)	
Age(years)				0.465
< 60	12 (40)	4 (30.8)	8 (47.1)	
≥ 60	18 (60)	9 (69.2)	9 (52.9)	
Sex				0.264
Male	18 (60)	6 (46.2)	12 (70.6)	
Female	12 (40)	7 (53.8)	5 (29.4)	
Lesion size (mm)				**0.002**
< 10	11 (36.7)	9 (69.2)	2 (11.8)	
≥ 10	19 (63.3)	4 (30.8)	15 (88.2)	
Location				0.971
Upper esophagus	2 (6.7)	1 (7.7)	1 (5.9)	
Middle esophagus	23 (76.7)	10 (76.9)	13 (76.5)	
Lower esophagus	5 (16.7)	2 (15.4)	3 (17.6)	
Color under white light endoscopy				0.167
Reddish	18 (60.0)	6 (46.2)	12 (70.6)	
Whitish	7 (23.3)	3 (23.1)	4 (23.5)	
No obvious change	5 (16.7)	4 (30.7)	1 (5.9)	
Gross type				0.379
Elevated	1 (3.3)	0 (0)	1 (5.9)	
Flat	25 (83.4)	12 (92.3)	13 (76.5)	
Depressed	4 (13.3)	1 (7.7)	3 (17.6)	
Pink color sign				0.613
Yes	4 (13.3)	1 (7.7)	3 (17.6)	
No	26 (86.7)	12 (92.3)	14 (82.4)	
IPCL				< **0.001**
Type A	10 (33.3)	10 (76.9)	0 (0)	
Type B	20 (66.7)	3 (23.1)	17 (100)	
Brownish color background				0.138
Yes	17 (56.7)	5 (38.5)	12 (70.6)	
No	13 (43.3)	8 (61.5)	5 (29.4)	
Number of biopsies				–
1	30 (100)	13 (100)	17 (100)	
≥ 2	0 (0)	0 (0)	0 (0)	

*p* value < 0.05 was highlighted by bold values

LGIN = low grade intraepithelial neoplasm, HGIN = high grade intraepithelial neoplasm, ESCC = esophageal squamous cell carcinoma, IPCL = intrapapillary capillary loops

### Histological upgrade to ESCC in biopsy diagnosed HGIN

**Fig. 2:**
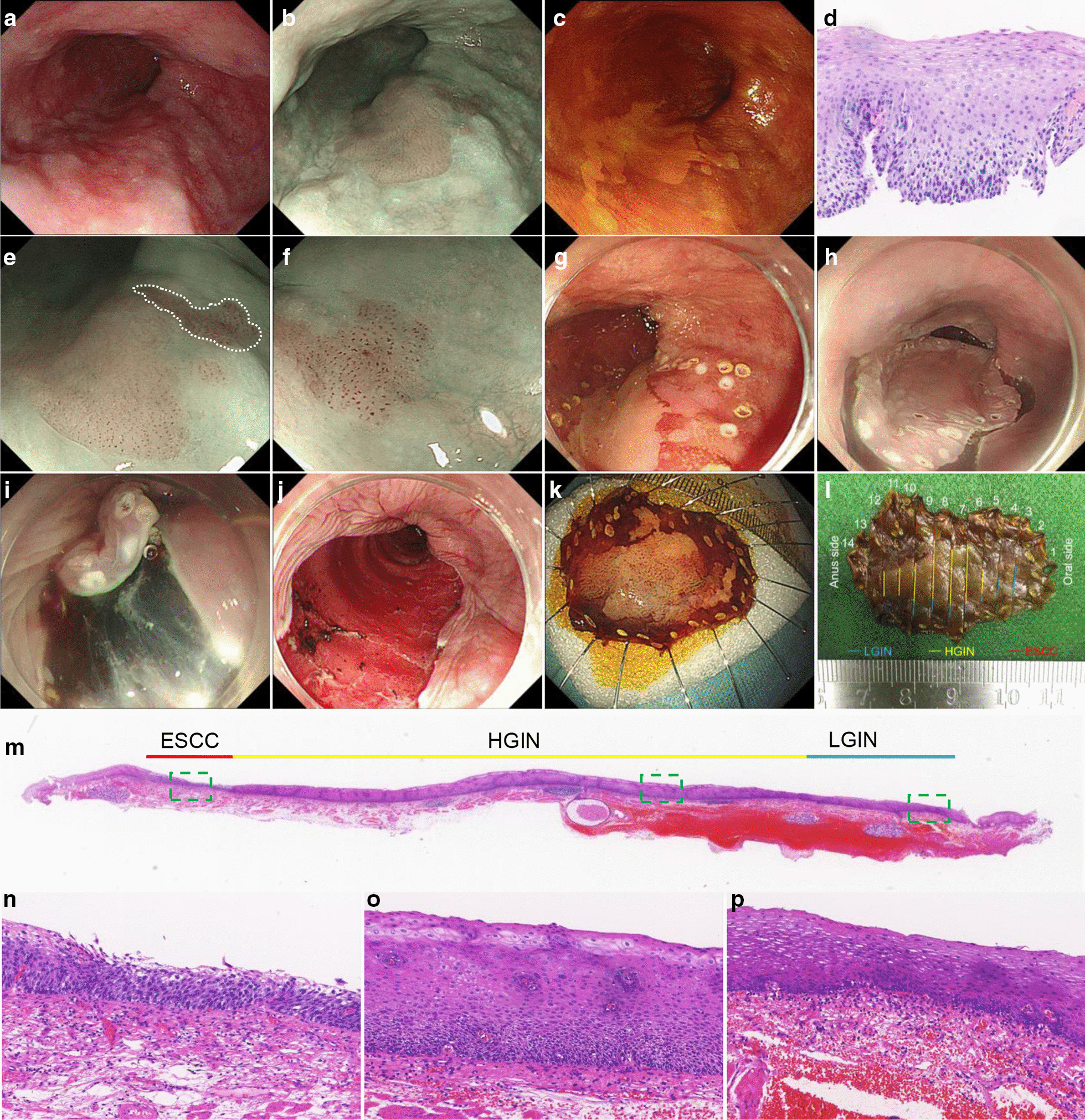
A patient who was diagnosed as HGIN on biopsy and subsequently upgraded to ESCC after resection. **a** A reddish flat lesion is observed in the middle esophagus; **b** the lesion is demarcated brownish area under narrow band imaging; **c** unstained area is observed after 1% iodine spraying. **d** One biopsy is taken from unstained area and is diagnosed as HGIN; **e** magnifying endoscopy shows type B1 IPCL; **f** higher irregular type B1 IPCL is observed within the area correspond to area (white dotted lines) in panel **e**; **g** pink color sign is positive in the area correspond to area (white dotted lines) in panel **e**; **h** circumferential incision was made after marking the lesions and submucosal injection; **i** submucosal dissection; **j** the lesion was completely removed; **k** resected specimen after 1% iodine spraying; **l** pathological diagnosed as esophageal squamous cell carcinoma in situ coexists with HGIN and LGIN; **m** the histology of No.8 slides is shown in low-power view; and histology in high-power view correspond to box in panel **m** was shown in **n** carcinoma in situ; **o** HGIN; and **p** LGIN

Among the 83 lesions that were diagnosed as HGIN on biopsy, 34 were upgraded to ESCC after resection, 5 of which (5/83, 6.0%) were submucosal invasive cancers. Perforation occurred in three patients during the endoscopic procedure and were closed using clips, after which delayed bleeding occured in two patients. Six resections were non-curative due to indefinite or positive margins (n = 4), or deep submucosal invasion and vascular invasion (n = 3) (Additional file 2: Table S2). Histological upgrades to ESCC in biopsy-demonstrated HGIN was associated with lesion size (*p* = 0.001), location (*p* = 0.018), and pink color sign (*p* = 0.002), but not with age (*p* = 0.654), sex (*p* = 0.326), tumor color (*p* = 0.090), gross type (*p* = 0.073), IPCL (*p* = 0.266), background color (*p* = 0.101), or number of biopsies performed (*p* = 0.131) (Table 4). A representative case of biopsy-diagnosed HGIN upgraded to ESCC was is shown in Fig. 2.

**Table 4:** Risk factors associated with histological upgrade to ESCC in patients diagnosed with HGIN on biopsy

Variables	Total (n = 83)	Final histology		*p* value
		Non-ESCC (n = 49)	ESCC (n = 34)	
Age(years)				0.654
< 60	27 (32.5)	15 (30.6)	12 (35.3)	
≥ 60	56 (67.5)	34 (69.4)	22 (64.7)	
Sex				0.326
Male	56 (67.5)	31 (63.3)	25 (73.5)	
Female	27 (32.5)	18 (36.7)	9 (26.5)	
Lesion size (mm)				**0.001**
< 10	13 (15.7)	13 (26.5)	0 (0)	
10–20	27 (32.5)	17 (34.7)	10 (29.4)	
≥ 20	43 (51.5)	19 (38.8)	24 (70.6)	
Circumferential extent of lesions				0.159
< 50%	74 (89.2)	45 (91.8)	29 (85.3)	
50–75%	7 (8.4)	4 (8.2)	3 (8.8)	
> 75%	2 (2.4)	0 (0)	2 (5.9)	
Location				**0.018**
Upper esophagus	8 (19.7)	4 (8.2)	4 (11.8)	
Middle esophagus	65 (78.3)	43 (87.7)	22 (64.7)	
Lower esophagus	10 (12.0)	2 (4.1)	8 (23.5)	
Color				
Reddish	55 (66.3)	32 (65.3)	23 (67.6)	0.090
Whitish	15 (18.1)	8 (16.3)	7 (20.6)	
No obvious change	10 (12.0)	9 (18.4)	1 (2.8)	
Gross type				0.073
Elevated	2 (2.4)	0 (0)	2 (5.9)	
Flat	64 (77.1)	41 (83.7)	23 (67.6)	
Depressed	17 (20.5)	8 (16.3)	9 (26.5)	
Pink color sign				**0.002**
Yes	44 (53.0)	19 (38.8)	25 (73.5)	
No	39 (47.0)	30 (61.2)	9 (26.5)	
IPCL				0.266*
Type A	3 (3.6)	3 (6.1)	0 (0)	
Type B	80 (96.4)	46 (93.9)	34 (100)	
Brownish color background				0.101
Yes	66 (79.5)	36 (73.5)	30 (88.2)	
No	17 (20.5)	13 (26.5)	4 (11.8)	
Number of biopsies				0.131
1	61 (73.5)	39 (79.6)	22 (64.7)	
≥ 2	22 (26.5)	10 (20.4)	12 (35.3)	

*p* value < 0.05 was highlighted by bold values

HGIN = high grade intraepithelial neoplasm, ESCC = esophageal squamous cell carcinoma, IPCL = intrapapillary capillary loops

^*^Fisher’s exact test

## Discussion

Esophageal squamous cell neoplasia is thought to arise from the basal layer of the epithelium, with dysplastic cell growth that gradually extends to the full layer of epithelial cells, and infiltrating into the lamina propria mucosae or deeper. It is recommended that HGINs and superficial ESCCs with limited lymph node metastases be treated with endoscopic resection, and the endoscopic resected specimens be evaluated pathologically for curability. The risk of lymph node metastasis is closely associated with the depth of tumor invasion. Superficial ESCCs classified as carcinoma in situ or that invade the lamina propria are absolute indication for endoscopic resection due to the near-zero risk of lymph node metastasis [8, 9, 20]. ESCCs invading the muscularis mucosae or submucosal layer to 200 μm or less are relative indications [9, 20]. A meta-analysis including 2864 superficial ESCC patients showed that the probability of lymph node metastasis in lesions invading the muscularis mucosae and submucosal layer to 200 μm or less reached 8.8% and 23.2%, respectively [21]. The risk of lymph node metastasis is associated with vascular or lymphatic invasion. A Japanese study reported that the cumulative 5-year metastasis rates in patients with esophageal squamous cell mucosal cancer without lymphovascular involvement was 0.7% [22], which suggests that ESCC invading muscularis mucosae is still an indication for endoscopic resection if no lymphovascular invasion is observed [8]. In our center, endoscopic resection is recommended for patients with HGINs and superficial ESCCs without distant or lymph node metastases, excluding those with obvious submucosal invasion (SM2 or deeper invasion). Additional treatments are recommended when pathological finding indicate positive vertical margins, lymphovascular invasion, poor differentiated histology and submucosal invasion of more than 200 μm according to the Consensus of the Chinese Society of Gastroenterology [10]. In addition, patients with lesions involving more than 3/4 of the circumferential extent with a high risk of stricture after endoscopic resection were offered a sufficient explanation and discussion of the risks and postoperative preventative strategies prior to the operation (i.e., oral, or local steroids).

In routing practice, approximate biopsies are taken from suspected lesions to determine the histological category, and appropriate decisions are made regarding treatment. In our study, we found that the discrepancy rate between forceps biopsy and resected specimens was 45.3%, which was higher than previous data (34.5%, 29/84 lesions) from Korea [14]. This is likely because our study included cases of biopsies diagnosed as negative/indefinite for neoplasia. However, the discrepancy rate in our study was lower than the 79.2% (41.7% upstaged, 37.5% downstaged) that was published by scholars from the UK, likely due to the greater number of histological grade levels for cancer that were grouped in their study [13].

Many factors may contribute to the histological discrepancy between forceps biopsy and resected specimens. First, the pathological diagnostic criteria for squamous cell intraepithelial neoplasia are different from those of Western and Japanese pathologists, even among individual pathologist. Some lesions with stromal invasion in resected specimens may be diagnosed as HGIN, LGIN, or reactive atypical lesions on biopsy according to Western criteria [23]. In this study, the slides of biopsy and resected specimens were reviewed by two pathologists according to the fifth edition of the WHO classification of digestive tumors to reduce bias. Second, SESCN is a heterogeneous disease (Fig. 2), so small biopsy fragments are not sufficient to evaluate the pathology of the entire lesion. In addition, the poor tissue orientation of the biopsy also influences the grade of intraepithelial neoplasia [24]. Performing multiple biopsy specimens may increase the diagnostic yield, with six biopsies likely to achieve 100% diagnostic accuracy in esophageal carcinoma [25]. A concordant diagnosis of HGIN based on more than 4 biopsies was easier to achieve than that based on fewer biopsies [26]. However, while more biopsies is acceptable strategy for advanced ESCC, this may cause submucosal fibrosis in superficial lesions and increase the complexity of endoscopic resection. Park et al. [14] recommended one biopsy for superficial esophageal squamous cell neoplasia less than 14 mm and two biopsies for larger lesions. In our study, most of the lesions (74.5%) were assessed with less than 2 biopsies, and the number of biopsies and median tumor area per biopsy were not statistically associated with histological discrepancies. Moreover, some superficial lesions are covered by normalized epithelial cells, such as basal layer type ESCC, or mature epithelial (verrucous carcinoma), causing them to be underdiagnosed as LGIN on biopsy [27, 28]. Thus, to increase the accuracy of biopsy for superficial lesions, it is recommended that the biopsy site be in area with pink color sign or higher irregular IPCLs [14].

Squamous cell intraepithelial neoplasia is considered a precursor to ESCC. The risk of ESCC is related to the degree of dysplasia and has found to be 1.4%, 4.5% and 15.5% for mild, moderate, and severe dysplasia, respectively [29]. Li et al. applied endoscopic mucosal resection for esophageal squamous cell LGIN and none were upgraded to HGIN or ESCC, while 6 cases (9.2%) were upstaged to HGINs during follow-up [30]. In our study, we found that 17 of the 119 (14.3%) ESCC and HGIN cases were under-diagnosed as LGIN, or indefinite/negative for neoplasia. this may be corrected using magnifying endoscopy. The positive and false predictive value of type B IPCL for diagnosing HGIN and ESCC was 85.0% and 100.0% in biopsies diagnosed as negative/indefinite for neoplasia and LGIN, respectively. Meanwhile, a previous study revealed that magnifying endoscopy had higher diagnostic accuracy than biopsy for esophageal lesions [31]. The above data suggest that patients with esophageal lesions with type B IPCL, but not confirmed as HGIN or ESCC on the initial biopsy, should be receive endoscopic resection for pathological assessment or a repeat biopsy to avoid under-treatment.

While it has been believed that HGIN does not have the potential to metastasize to the lymph nodes or form distant metastases. the invasive foci may have been missed when the diagnosis of HGIN was made based on small endoscopic biopsy fragments. Shimizu et al. [32] reported that more than 30% of biopsies showed that diagnosed HGIN was an invasive carcinoma. Similarly, we found that 34 of the 83 (41%) biopsies diagnosed as HGIN were invasive cancers, even to the submucosal layer (5/83, 6.0%). Histological upgrade to ESCC in biopsy-diagnosed HGIN was associated with lesion size, location, and pink color sign. In addition, a Chinese study found that 19 of the 169 biopsy-diagnosed HGIN cases (11.24%) had lesions infiltrating the submucosa after resection, and the risk factors were lesion size > 2 cm, depressed and excavated patterns, and ≤ 4 biopsy samples [26]. However, local treatment, such as endoscopic resection as a “complete biopsy” or alternative therapies including radiofrequency ablation [33] and photodynamic therapy [34] can be applied for HGIN. A high proportion of biopsy-diagnosed HGIN lesions were later diagnosed as carcinoma, therefore, carefully selecting patients based on the invasive depth of the lesions is essential for successful treatment of biopsy diagnosed HGINs. Additionally, the invasive depth of the lesions should be judged carefully by the morphology, endoscopic ultrasound and magnifying endoscopy prior to treatment [35]. For lesions suspected to have deep submucosal invasion, a repeat biopsy should be considered to correct the initial histological category of the biopsy.

There are a few limitations to this study. First, due to nature of this single-center retrospective study, there is a potential for selection bias. Second, the number of cases that were diagnosed as indefinite for neoplasia and LGIN on biopsy was small, since most patients without pink color sign or type B IPCLs were managed by surveillance. Therefore, our results should be validated in a large prospective study conducted in multiple centers.

## Conclusions

Forceps biopsy is not sufficient for accurate determination of the final histology of SESCN. For lesions that are diagnosed as indefinite for neoplasia or LGIN on biopsy, type B IPCL and tumor size > 10 mm were risk factors for histological upgrade to higher categories, and therefore, re-biopsy or endoscopic resection should be undertaken to avoid underdiagnosis. For lesions that are diagnosed as HGIN or ESCC on biopsy, especially HGIN with risk factors, invasive depth should be evaluated carefully before endoscopic resection to avoid over treatment.

## Supplementary Information

**Additional file 1: Table S1.** Clinicopathological factors associated with histological discrepancy in patients with superficial esophageal squamous cell neoplasia.

**Additional file 2: Table S2.** Cases with no-curation resection in biopsy demonstrated HGINs.

## Data Availability

The datasets used and/or analyzed during the current study are available from the corresponding author on reasonable request.

## Abbreviations

ESD: Endoscopic submucosal dissection
MBM: Multiband mucosectomy
LGIN: Low-grade intraepithelial neoplasia
HGIN: High-grade intraepithelial neoplasia
ESCC: Esophageal squamous cell carcinoma
SESCN: Superficial esophageal squamous cell neoplasia
IPCL: Intrapapillary capillary loops
WHO: World Health Organization
SD: Standard deviation
IQR: Interquartile ranges

## References

[1] Bray F, Ferlay J, Soerjomataram I, Siegel RL, Torre LA, Jemal A (2018). Global cancer statistics 2018: GLOBOCAN estimates of incidence and mortality worldwide for 36 cancers in 185 countries. CA Cancer J Clin.

[2] Arnold M, Soerjomataram I, Ferlay J, Forman D (2015). Global incidence of oesophageal cancer by histological subtype in 2012. Gut.

[3] Zeng H, Zheng R, Zhang S, Zuo T, Xia C, Zou X, Chen W (2016). Esophageal cancer statistics in China, 2011: estimates based on 177 cancer registries. Thorac Cancer.

[4] Gao QY, Fang JY (2015). Early esophageal cancer screening in China. Best Pract Res Clin Gastroenterol.

[5] Chen R, Liu Y, Song G, Li B, Zhao D, Hua Z, Wang X, Li J, Hao C, Zhang L et al. Effectiveness of one-time endoscopic screening programme in prevention of upper gastrointestinal cancer in China: a multicentre population-based cohort study. Gut 2020. 10.1136/gutjnl-2019-320200 PMC7815635 32241902

[6] Dixon MF (2002). Gastrointestinal epithelial neoplasia: Vienna revisited. Gut.

[7] Ishihara R, Arima M, Iizuka T, Oyama T, Katada C, Kato M, Goda K, Goto O, Tanaka K, Yano T (2020). Endoscopic submucosal dissection/endoscopic mucosal resection guidelines for esophageal cancer. Dig Endosc.

[8] Park CH, Yang DH, Kim JW, Kim JH, Kim JH, Min YW, Lee SH, Bae JH, Chung H, Choi KD (2020). Clinical practice guideline for endoscopic resection of early gastrointestinal cancer. Clin Endosc.

[9] Pimentel-Nunes P, Dinis-Ribeiro M, Ponchon T, Repici A, Vieth M, De Ceglie A, Amato A, Berr F, Bhandari P, Bialek A (2015). Endoscopic submucosal dissection: European Society of Gastrointestinal Endoscopy (ESGE) Guideline. Endoscopy.

[10] Chinese Society of Gastroenterology (2016). Consensus on screening, diagnosis and treatment of early esophageal squamous cell carcinoma and precancerous lesions in China (Beijing, 2015). Zhong Hua Xiao Hua Nei Jing Za Zhi.

[11] Wang Z, Lu H, Wu L, Yuan B, Liu J, Shi H, Wang F (2016). Long-term outcomes of endoscopic multiband mucosectomy for early esophageal squamous cell neoplasia: a retrospective, single-center study. Gastrointest Endosc.

[12] Jin XF, Sun QY, Chai TH, Li SH, Guo YL (2013). Clinical value of multiband mucosectomy for the treatment of squamous intraepithelial neoplasia of the esophagus. J Gastroenterol Hepatol.

[13] Kuan JY, Baskind S, Kim Y, McGrath S, Chaparala R, Assadsangabi A, Prasad N, Regi G, Ang Y (2020). Endoscopic resection of early squamous neoplasia of the oesophagus: long-term follow-up in a UK population from a tertiary hospital. Eur J Gastroenterol Hepatol.

[14] Park YJ, Kim GH, Park DY, Lee S, Lee MW, Lee BE, Song GA (2019). Histopathologic discrepancies between endoscopic forceps biopsy and endoscopic resection specimens in superficial esophageal squamous neoplasms. J Gastroenterol Hepatol.

[15] WHO Classification of Tumours Editorial Board. Digestive System Tumours. 5th Edition. Volume 1.

[16] Yang L, Jin P, Wang X, Zhang T, He YQ, Zhao XJ, Li N, Yang GZ, Sheng JQ (2018). Risk factors associated with histological upgrade of gastric low-grade dysplasia on pretreatment biopsy. J Dig Dis.

[17] Jung JI, Kim GH, Hoseok I, Park DY, Kim TK, Cho YH, Sung YW, Choi MK, Lee BE, Song GA (2014). Clinicopathologic factors influencing the accuracy of EUS for superficial esophageal carcinoma. World J Gastroenterol.

[18] Ishihara R, Kanzaki H, Iishi H, Nagai K, Matsui F, Yamashina T, Matsuura N, Ito T, Fujii M, Yamamoto S (2013). Pink-color sign in esophageal squamous neoplasia, and speculation regarding the underlying mechanism. World J Gastroenterol.

[19] Minami H, Isomoto H, Inoue H, Akazawa Y, Yamaguchi N, Ohnita K, Takeshima F, Hayashi T, Nakayama T, Nakao K (2014). Significance of background coloration in endoscopic detection of early esophageal squamous cell carcinoma. Digestion.

[20] Kitagawa Y, Uno T, Oyama T, Kato K, Kato H, Kawakubo H, Kawamura O, Kusano M, Kuwano H, Takeuchi H (2019). Esophageal cancer practice guidelines 2017 edited by the Japan esophageal society: part 2. Esophagus.

[21] Xu W, Liu XB, Li SB, Yang ZH, Tong Q (2020). Prediction of lymph node metastasis in superficial esophageal squamous cell carcinoma in Asia: a systematic review and meta-analysis. Dis Esophagus.

[22] Yamashina T, Ishihara R, Nagai K, Matsuura N, Matsui F, Ito T, Fujii M, Yamamoto S, Hanaoka N, Takeuchi Y (2013). Long-term outcome and metastatic risk after endoscopic resection of superficial esophageal squamous cell carcinoma. Am J Gastroenterol.

[23] Itabashi M, Nasierowska-Guttmejer A, Shimoda T, Majewski P, Rezner W, Sikora K, Srutek E, Steplewska K, Swatek J, Szumilo J (2017). The importance of the concept and histological criteria of "intraepithelial squamous cell carcinoma" of the esophagus: in comparison between Western and Japanese criteria. Esophagus.

[24] Savant D, Zhang Q, Yang Z. Squamous neoplasia in the esophagus. Arch Pathol Lab Med 2020. 10.5858/arpa.2020-0058-RA 32271610

[25] Lal N, Bhasin DK, Malik AK, Gupta NM, Singh K, Mehta SK (1992). Optimal number of biopsy specimens in the diagnosis of carcinoma of the oesophagus. Gut.

[26] Sang HM, Cao JL, Soyfoo MD, Zhang WM, Jiang JX, Xu SF (2019). Endoscopic and histopathology characteristics in patients with esophageal high-grade intraepithelial neoplasia. Dig Surg.

[27] Shimizu Y, Yoshida T, Kato M, Hirota J, Ono S, Nakagawa M, Kobayashi T, Kubota K, Asaka M (2010). Low-grade dysplasia component in early invasive squamous cell carcinoma of the esophagus. J Gastroenterol Hepatol.

[28] Matkovic E, Schwalbe M, Matkowskyj KA (2016). Pathologic features of esophageal and gastric malignancies. Cancer Treat Res.

[29] Wei WQ, Hao CQ, Guan CT, Song GH, Wang M, Zhao DL, Li BY, Bai WL, Hou PY, Wang JW (2020). Esophageal histological precursor lesions and subsequent 8.5-year cancer risk in a population-based prospective study in China. Am J Gastroenterol.

[30] Li H, Cheng JL, Dong NN, Cui CY, Diao TY, Zhou XR (2013). Effects of endoscopic mucosal resection in patients with low-grade intraepithelial dysplasia of esophageal squamous cells. Dig Surg.

[31] Nagai K, Ishihara R, Ishiguro S, Ohta T, Kanzaki H, Yamashina T, Aoi K, Matsuura N, Ito T, Fujii M (2014). Endoscopic optical diagnosis provides high diagnostic accuracy of esophageal squamous cell carcinoma. BMC Gastroenterol.

[32] Shimizu Y, Kato M, Yamamoto J, Ono Y, Katsurada T, Ono S, Mori Y, Nakagawa M, Nakagawa S, Itoh T (2006). Histologic results of EMR for esophageal lesions diagnosed as high-grade intraepithelial squamous neoplasia by endoscopic biopsy. Gastrointest Endosc.

[33] Lei S, Mulmi Shrestha S, Shi R (2020). Radiofrequency ablation for early superficial flat esophageal squamous cell neoplasia: a comprehensive review. Gastroenterol Res Pract.

[34] Hua X, Li Y, Ma H, Zhang W, Qin J, Zhang J, Cao H (2017). Photodynamic therapy versus endoscopic submucosal dissection for management of patients with early esophageal neoplasia: a retrospective study. J Thorac Dis.

[35] Shimamura Y, Ikeya T, Marcon N, Mosko JD (2017). Endoscopic diagnosis and treatment of early esophageal squamous neoplasia. World J Gastrointest Endosc.

