# Supplementary material for: Endoscopic resections for superficial esophageal squamous cell epithelial neoplasia: focus on histological discrepancies between biopsy and resected specimens
Source: BMC Gastroenterol. 2021 Mar 9;21:114. doi: 10.1186/s12876-021-01694-9 (PMC7941920; doi:10.1186/s12876-021-01694-9)
Supplement: Supplementary file 1 — Additional file 1: Table S1. Clinicopathological factors associated with histological discrepancy in patients with superficial esophageal squamous cell neoplasia. [file 12876_2021_1694_MOESM1_ESM.docx]

Table S1 Clinicopathological factors associated with histological discrepancy in patients with superficial esophageal squamous cell neoplasia

| Variables | Total  (n=137) | Accordant  (n=75) | Discordant  (n=62) | *p* value |
| --- | --- | --- | --- | --- |
| Age(years) |  |  |  | 0.128 |
| <60 | 46(33.6) | 21(28.0) | 25(40.3) |  |
| ≥60 | 91(66.4) | 54(72.0) | 37(59.7) |  |
| Sex |  |  |  | 0.550 |
| Male | 92(67.2) | 52(69.3) | 40(64.5) |  |
| Female | 45(32.8) | 23(30.7) | 22(35.5) |  |
| Maximum lesion size (mm) |  |  |  | 0.599 |
| <10 | 27(19.7) | 16(21.3) | 11(17.7) |  |
| ≥10 | 110(80.3) | 59(78.7) | 51(82.3) |  |
| Location |  |  |  | 0.086 |
| Upper esophagus | 13(9.5) | 7(9.3) | 6(9.7) |  |
| Middle esophagus | 105(76.6) | 62(82.7) | 43(69.4) |  |
| Lower esophagus | 19(13.9) | 6(8.0) | 13(20.9) |  |
| Color | | |  | 0.356 |
| Reddish | 99(72.3) | 55(73.4) | 44(71.0) |  |
| Whitish | 23(16.8) | 10(13.3) | 13(20.9) |  |
| No obvious change | 15(10.9) | 10(13.3) | 5(8.1) |  |
| Gross type |  |  |  | 0.353 |
| Elevated | 11(8.0) | 8(10.7) | 3(4.8) |  |
| Flat | 97(70.8) | 50(66.7) | 47(75.8) |  |
| Depressed | 29(21.2) | 17(22.6) | 12(19.4) |  |
| Pink color sign |  |  |  | 0.791 |
| Yes | 68(49.6) | 38(50.7) | 30(48.4) |  |
| No | 69(50.4) | 37(49.3) | 32(51.6) |  |
| IPCL |  |  |  | 0.945 |
| Type A | 13(9.5) | 7(9.3) | 6(9.7) |  |
| Type B | 124(90.5) | 68(90.7) | 56(90.3) |  |
| Brownish color background | |  |  | 0.833 |
| Yes | 105(76.6) | 58(77.4) | 47(75.8) |  |
| No | 32(23.4) | 17(22.6) | 15(24.2) |  |
| Number of biopsies |  |  |  | 0.364 |
| 1 | 102(74.5) | 53(70.7) | 49(79.1) |  |
| ≥2 | 35(25.5) | 22(29.3) | 13(20.9) |  |
| Area of biopsy tissue, mm^2^ (Mean ± SD^)^ | 2.95±1.57 | 2.77±1.56 | 3.14±1.57 | 0.261 |
| Median tumor area, mm^2^ (IQR) | 169.6 (60.8-471.0) | 173.5 (56.5-431.8) | 155.4 (65.2-471.0) | 0.782 |
| Median tumor area/biopsy number, mm^2^ (IQR) | 141.3 (57.7-329.7) | 141.3 (55.0-270.8) | 148.4 (64.3-417.0) | 0.556 |
| Time gap between biopsy and resection (days) | 19(13-37) | 19(13-38) | 18 (13-36) | 0.654 |
| Histological category of the biopsy |  |  |  | **<0.001** |
| Category 1/2/3 | 30(21.9) | 8(10.7) | 22(35.5) |  |
| Category 4 | 83(60.6) | 45(60.0) | 38(61.3) |  |
| Category 5 | 24(17.5) | 22(29.3) | 2(3.2) |  |

IPCL = intrapapillary capillary loops; SD = standard deviation; IQR = interquartile ranges
