# Supplementary material for: Endoscopic resections for superficial esophageal squamous cell epithelial neoplasia: focus on histological discrepancies between biopsy and resected specimens
Source: BMC Gastroenterol. 2021 Mar 9;21:114. doi: 10.1186/s12876-021-01694-9 (PMC7941920; doi:10.1186/s12876-021-01694-9)
Supplement: Supplementary file 2 — Additional file 2: Table S2. Cases with no-curation resection in biopsy demonstrated HGINs. [file 12876_2021_1694_MOESM2_ESM.docx]

Table S2 cases with no-curation resection in biopsy demonstrated HGINs

| No. | Age group | Sex | Location | Gross type | Size(mm) | IPCL | Final pathology | En bloc | Margin (lateral/vertical) | Invasive depth | Vascular invasion |
| --- | --- | --- | --- | --- | --- | --- | --- | --- | --- | --- | --- |
| 1 | 60-70 | 1 | Middle | 2b | 28 | B1 | SCC | No | indefinite/negative | M2 | negative |
| 2 | 50-60 | 1 | Lower | 2c | 35 | B1 | SCC | No | positive/negative | M2 | negative |
| 3 | 50-60 | 1 | Middle | 2b | 38 | B1 | SCC | Yes | positive/negative | M2 | negative |
| 4 | 80-90 | 1 | Middle | 2b | 40 | B1 | SCC | Yes | negative/indefinite | SM2 | negative |
| 5 | 60-70 | 2 | Middle | 2b | 40 | B2 | SCC | Yes | negative/negative | SM2 | positive |
| 6 | 60-70 | 1 | Middle | 2b | 100 | B1 | SCC | Yes | negative/negative | SM2 | negative |
